# Transcatheter Aortic Valve Replacement Incidentalomas: A Multimodality Imaging Case of Giant Right Coronary Aneurysm

**DOI:** 10.1155/2018/9738530

**Published:** 2018-03-26

**Authors:** Jamal Janjua, Esosa G. Odigie-Okon, Premnauth Rabindranauth, Richard J. Wittchow, Aiman Riaz

**Affiliations:** ^1^Department of Internal Medicine, Gundersen Health System, La Crosse, WI 54601, USA; ^2^Department of Cardiology, Gundersen Health System, La Crosse, WI 54601, USA; ^3^Department of Cardiothoracic Surgery, Gundersen Health System, La Crosse, WI 54601, USA; ^4^Department of Pathology, Gundersen Health System, La Crosse, WI 54601, USA

## Abstract

Giant coronary artery aneurysms (CAAs) are defined as having a diameter of greater than 2 cm. We report a case of an 82-year-old male with severe aortic stenosis incidentally diagnosed with giant right coronary artery aneurysm (gRCAA) while undergoing evaluation for transcather aortic valve replacement (TAVR). It was causing a mass effect on the right cardiac chambers but was otherwise asymptomatic. Our patient was successfully treated with surgical excision of aneurysm with concomitant coronary artery bypass grafting (CABG) and surgical aortic valve replacement (SAVR). The patient remained stable at discharge and on serial follow-ups for two years. In conclusion, due to the associated complication and increased risk of mortality with giant coronary aneurysms, we recommend surgical approach instead of medical management alone. We also call for evidence-based recommendations and guidelines for management of TAVR incidentalomas.

## 1. Introduction

Transcatheter aortic valve replacement (TAVR) has been proven to be a safe and effective minimally invasive procedure for aortic stenosis and has been shown to reduce mortality among high-risk patients with aortic valve stenosis irrespective of their candidacy for surgical replacement, typically octogenarians and nonagenarians [1]. As a result of patients' advanced age, incidentalomas ranging from intracardiac thrombi, extracardiac malignancies and congenital heart diseases are frequently encountered and alter the surgical plan. Coronary artery aneurysm (CAA) is a rare entity typically diagnosed incidentally through imaging studies or at postmortem. Giant coronary artery aneurysms (gCAAs) are even rarer and are defined as having a diameter more than 2 cm. It can be broadly classified into congenital or acquired (e.g., iatrogenic, atherosclerosis, and infectious). We present a multimodality imaging case of a giant right coronary artery aneurysm (gRCAA) found incidentally during workup for aortic valve replacement and outline the unique challenge it presented in this setting.

## 2. Case Report

**Figure 1:**
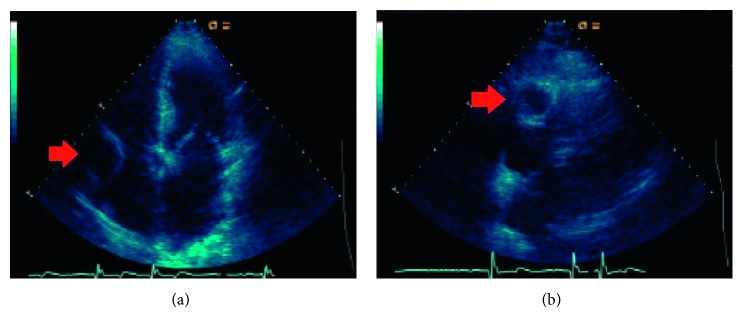
(a, b) 2D Echocardiogram showing echolucent mass in the right AV groove with mass effect. AV: atrioventricular.

**Figure 2:**
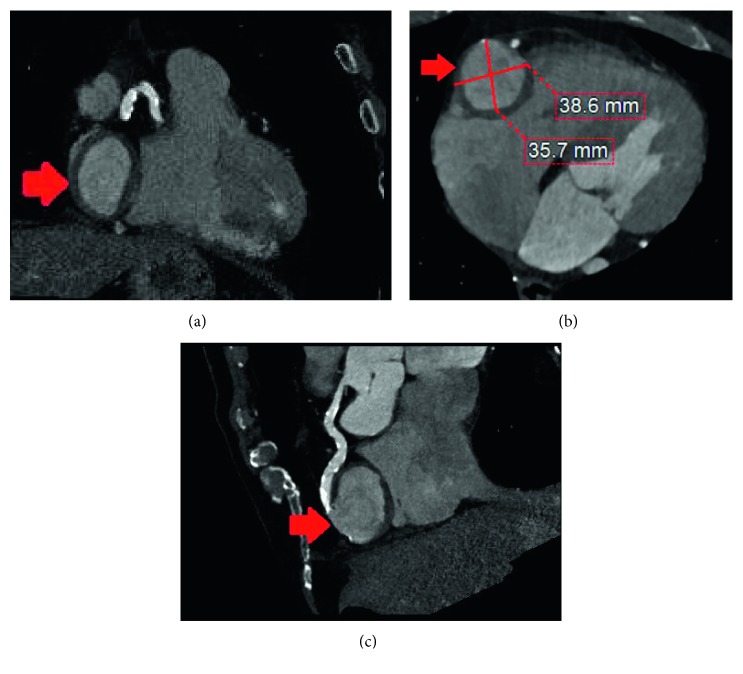
CT coronary angiogram. Thrombosed RCA aneurysm in coronal (a) and axial (b) view in right AV groove. (c) Curved multiplanar reformatted image displaying full length of the RCA. AV: atrioventricular; RCA: right coronary artery.

**Figure 3:**
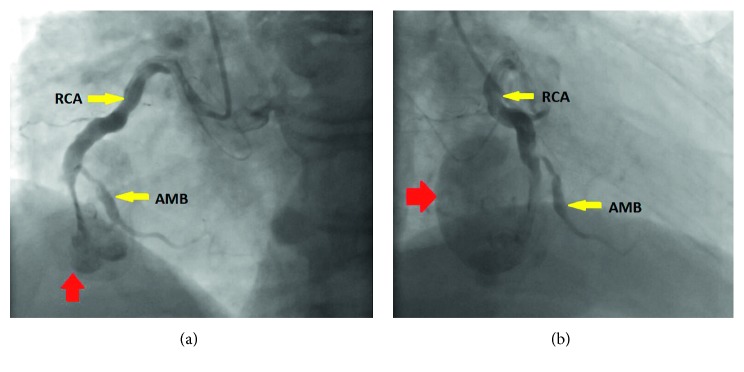
(a, b) Invasive coronary angiogram showing thrombosed RCA aneurysm with contrast and no distal flow. AMB: acute marginal branch; RCA: right coronary artery.

**Figure 4:**
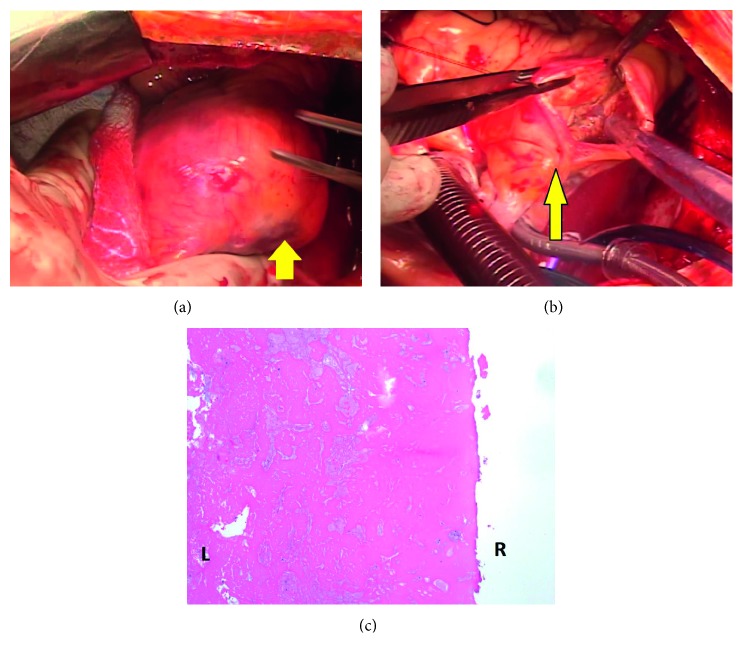
Intraoperative surgeons' view of the RCA aneurysm intact (a) and excised (b). (c) Photomicrographs of the fibrin thrombus removed from the RCA aneurysm taken at 40x magnification. Shown is the more mature portion of the thrombus on the right side composed of dense, laminated fibrin, while the luminal side (left) has more recent thrombus deposition composed of blood/platelets alternating with fibrin bands (lines of Zahn). RCA: right coronary artery.

An 82-year-old male with symptomatic severe aortic stenosis, permanent atrial fibrillation, ascending aorta enlargement, and obstructive sleep apnea, was referred to our institution. Retrospectively, a transthoracic echocardiogram (TTE) performed 4 years prior had demonstrated an echolucent mass external to the heart located in the right atrioventricular junction causing mass effect on the right atrium and ventricle (Figure 1); however, it was not investigated further. Computed tomography angiogram (CTA) of the aorta was performed in consideration for TAVR based on his high-risk status as well as patient not wanting to undergo surgical aortic valve replacement (SAVR) for personal reasons. It showed a large encapsulated spherical mass with wall thickening in the right atrioventricular groove measuring 38.6 mm by 47.4 mm, continuous with the right coronary artery in the mid-occluded mid-right coronary artery (RCA) segment, findings consistent with a thrombosed gRCAA (Figures 2(a)–2(c)). Coronary angiography confirmed a totally occluded RCA with a mid-RCA dilation that demonstrated contrast swirling in the aneurysm (Figures 3(a) and 3(b)). The left coronary arterial system was without significant disease. The heart team including the cardiologists and cardiothoracic surgeons agreed that TAVR does not address his gRCAA, which could potentially be fixed by coiling, but this would likely lead to complications from the aneurysm including an RCA infarction, fistula, or other fatal complication intraoperatively. As such, he underwent a surgical aortic valve replacement with a 27 mm Sorin Solo Smart tissue valve, coronary artery bypass grafting was done using a saphenous vein graft to the distal posterior descending artery with end to side to acute marginal anastomosis, the gRCAA was excised and repaired with over sew of proximal and distal coronary artery (Figures 4(a)–4(c)) with a left atrial appendage amputation for his atrial fibrillation. Postoperative course was uneventful, and he was discharged home in a stable condition.

## 3. Discussion

Coronary artery aneurysm is defined as dilation of greater than 1.5 times than the reference vessel. It is rare with incidence of 0.02–0.04% in the general population [2]. Increased incidence, however, is reported in patients undergoing diagnostic procedures like angiography, ranging from 1.1 to 4.9% [3]. It usually involves the right coronary artery. In a study by Keyser et al., right-sided gCAA was found in 89% of the cases [4]. One of the most common childhood conditions associated with CAAs is Kawasaki disease, but it can also be found in connective tissue diseases (e.g., Marfan's and Ehlers-Danlos syndrome), infections (e.g., syphilis), inflammation (e.g., polyarthritis nodosa and systemic lupus erythematosus), and iatrogenic causes (like coronary angiography and stenting). The most common acquired cause of CAA is atherosclerosis as suggested in studies by Makris et al. [5] and Hartnell et al. [6] in as early as 1976 and 1985, respectively. The dreaded complications include rupture of CAA [2], thrombosis, embolization and myocardial infarction [7], congestive heart failure, and fistulous connections [8] among others.

Management remains controversial. Recommended surgical treatment involves resection of aneurysm with or without coronary bypass graft [2]. Other options include patch repair, coil embolization, and stents [9]. More recently, percutaneous techniques have been used as well [10]. Dai et al. reported a case of gRCAA which was successfully treated percutaneously with multiple overlapping stents [11]. Conservative management includes statins and antiplatelet agents or anticoagulation due to the fear of thrombus formation and distal embolization [12] although evidence is still lacking. In our patient, no evidence for vasculitis, infection or connective tissue disease was found. CT angiogram demonstrated significant calcific atherosclerotic coronary artery disease allowing us to positively confirm atherosclerosis as the most likely etiology.

Since our patient had aneurysm that remained asymptomatic for the past 4 years, one approach could have been to manage it conservatively. It was supported by the data from coronary artery surgery study (CASS) registry which followed patients throughout the 1980s, and no aneurysmal ruptures were found [13]. However, the authors of this paper propose the individualization of treatment based on patient-related factors and preferences. In our case, the patient required aortic valve replacement and had significant coronary artery disease in addition to the mass effect caused by the gCAA, so it made sense to address it at time of valve repair. An isolated, asymptomatic gCAA without valvular stenosis requiring intervention would have steered us in the direction of medical management alone.

## 4. Conclusion

Giant coronary aneurysm is a rare entity. However, the probability of encountering it in geriatric cardiology clinical practice is likely to increase with rising rates of percutaneous cardiac interventions like TAVR. With the increasing expansion of TAVR over SAVR to intermediate risk and possibly low-risk patients in the near future, percutaneous options for management of these pathologies will be of paramount importance to justify the eventual cost of TAVR over SAVR for these patients with otherwise limited life expectancies. In addition, knowledge of the various etiologies, associated complications, and treatment options of these incidentalomas are paramount. Optimal management option of GCA is still controversial, but we recommend surgical excision especially if aneurysm is causing a mass effect as seen in our patient. Most incidentalomas found during TAVR evaluation may be benign but can also represent lesions requiring therapeutic interventions that would otherwise not be necessary based on the patient's life expectancy, thus leading to more expensive medical costs. The specific relevance and implications of the pathologies will vary for the interventional cardiologist as well as the cardiothoracic surgeon. There is therefore a growing need for evidence-based recommendations and guidelines for management of TAVR incidentalomas.
